# LEGS: Visual Localization Enhanced by 3D Gaussian Splatting

**DOI:** 10.3390/jimaging12020084

**Published:** 2026-02-16

**Authors:** Daewoon Kim, I-gil Kim

**Affiliations:** Tech. Innovation Group, KT Corporation, 151, Taebong-ro, Seocho-gu, Seoul 06763, Republic of Korea; daewoon.kim@kt.com

**Keywords:** visual localization, camera pose estimation, Structure-from-Motion (SfM), Novel View Synthesis (NVS), synthetic view augmentation, 3D Gaussian Splatting (3DGS)

## Abstract

Accurate six-degree-of-freedom (6-DoF) visual localization is a fundamental component for modern mapping and navigation. While recent data-centric approaches have leveraged Novel View Synthesis (NVS) to augment training datasets, these methods typically rely on uniform grid-based sampling of virtual cameras. Such naive placement often yields redundant or weakly informative views, failing to effectively bridge the gap between sparse, unordered captures and dense scene geometry. To address these challenges, we present LEGS (Visual **L**ocalization **E**nhanced by 3D **G**aussian **S**platting), a trajectory-agnostic synthetic-view augmentation framework. LEGS constructs a joint set of 6-DoF camera pose proposals by integrating a coarse 3D lattice with the Structure-from-Motion (SfM) camera graph, followed by a visibility-aware, coverage-driven selection strategy. By utilizing 3D Gaussian Splatting (3DGS), our framework enables high-throughput, scene-specific synthesis within practical computational budgets. Experiments on standard benchmarks and an in-house dataset demonstrate that LEGS consistently improves pose accuracy and robustness, particularly in scenarios characterized by sparse sampling and co-located viewpoints.

## 1. Introduction

Accurate 6-DoF visual localization is a fundamental component in many modern systems such as mapping, augmented reality (AR), and robotic navigation. However, real-world image collections rarely follow the continuous and clean video trajectories often assumed in research datasets. Instead, captured images are typically unordered, with irregular camera layouts and multiple viewpoints sharing similar positions but different orientations.

In visual localization, recent work increasingly adopts deep learning-based formulations—e.g., direct pose regression and scene-coordinate prediction—because of their scalability and learned generalization [1,2,3,4,5,6]. Yet these methods, like structure-based pipelines, remain sensitive to dataset artifacts such as uneven viewpoint distributions, limited spatial coverage, and orientation bias, which commonly arise in unordered, RGB-only captures. This has motivated data-centric remedies that densify training views while respecting scene geometry, exemplified by LENS, which leverages NVS for localization dataset augmentation [7].

The core limitation of current visual localization lies in viewpoint imbalance. When images are captured along restricted trajectories or share similar headings, the resulting coverage of the scene becomes uneven. This leads to redundant or geometrically weak viewpoints, which in turn degrade triangulation quality and limit the overall benefit of synthetic augmentation. Recent work, such as LENS [7], showed that augmenting pose-regression datasets with spatially balanced synthetic views generated via NVS can partially alleviate this issue and improve localization accuracy. However, uniform grid sampling and scene-agnostic placement remain prone to occlusions and uninformative views, and high-quality rendering can be computationally expensive.

Overcoming this imbalance requires reasoning explicitly about scene geometry, visibility, and baseline diversity when generating new viewpoints. Motivated by this observation, we develop a visibility- and baseline-aware pose proposal-and-selection framework that systematically balances coverage while maintaining geometric consistency, enabling more effective and data-efficient synthetic-view augmentation.

Building on this concept, we propose LEGS, a trajectory-agnostic framework for viewpoint augmentation that efficiently proposes and filters realistic virtual camera poses in SE(3). LEGS combines a coarse 3D lattice with the SfM camera graph [8] to propose pose candidates, then selects a subset using a visibility-aware coverage-driven selection strategy constrained by baseline distance and angle. This design anchors proposals to the underlying scene geometry and visibility derived from SfM while maintaining sufficient parallax for effective triangulation.

LEGS further leverages the rendering efficiency of 3DGS [9], an explicit 3D scene representation that supports real-time, visibility-aware synthesis. This makes large-scale, geometry-informed view generation practical within limited computational budgets—an essential capability for producing diverse and informative training datasets.

Experiments on standard benchmarks such as Cambridge Landmarks [1] and an in-house dataset demonstrate that LEGS consistently improves pose accuracy and robustness across diverse conditions, including sparse sampling, orientation imbalance, and co-located viewpoints. The observed gains stem from the proposed pose generation and selection mechanism rather than specific network architectures.

In summary, LEGS offers a simple yet effective recipe for improving visual localization—combining geometry-informed camera pose generation with fast 3DGS synthesis—to deliver synthetic datasets that are both computationally efficient and physically meaningful.

## 2. Related Works

### 2.1. Structure from Motion

SfM estimates camera poses and reconstructs a sparse 3D structure from unordered image collections. It has become the standard foundation for building visual localization datasets. A typical SfM pipeline includes feature detection, feature matching, camera registration (incremental or global), and bundle adjustment. Among open-source implementations, COLMAP [8] is the most widely used reference system. Its public documentation and standardized output format (e.g., cameras.bin, images.bin, points3D.bin) have become a de facto common language for many localization benchmarks and toolkits. In practice, researchers often reconstruct scenes using COLMAP, export the resulting models, and use them for both training and evaluating localization systems.

Recent advances have improved the robustness and scalability of SfM by integrating learning-based components. Modern pipelines employ learned keypoint detectors and descriptors such as SuperPoint [10], and learned feature matchers such as SuperGlue [11] and LightGlue [12]. On top of these, hloc [13] provides a hierarchical, coarse-to-fine localization framework that couples global retrieval with local matching and pragmatic SfM registration, and has become a widely-adopted reference for large-scale benchmarks. Beyond modular pipelines, recent end-to-end or deeply integrated SfM systems such as VGGSfM [14] and MASt3R-SfM [15] leverage strong learned priors to stabilize pairwise geometry, improve correspondence quality, and reduce failure modes in challenging scenes. In parallel, VGGT [16] introduces a visual-geometry-grounded transformer that serves as a high-capacity backbone for multi-view geometry and can be plugged into SfM/localization stacks to further boost robustness.

Nevertheless, the accuracy of SfM still depends strongly on image overlap, viewpoint diversity, and surface texture. Sparse or orientation-biased captures can lead to incomplete reconstructions and poor visibility, motivating the need for data augmentation and viewpoint selection before localization. For this reason, SfM is not only a tool for reconstruction but also serves as a dataset generator: most widely used localization datasets either include or can be reconstructed from SfM models, and many evaluation pipelines assume COLMAP-compatible input and output formats.

### 2.2. Visual Localization

Visual localization methods can be broadly categorized into structure-based and learning-based approaches. Structure-based methods rely on geometric reasoning over 3D reconstructions obtained from SfM, whereas learning-based methods infer camera poses directly or indirectly from image evidence. In recent years, the field has shifted overwhelmingly toward learning-based formulations due to their scalability, adaptability, and potential to generalize across diverse environments.

Among learning-based approaches, absolute pose regression (APR) predicts a 6-DoF pose directly from a single image [1,2], while scene-coordinate regression (SCR) estimates per-pixel 3D coordinates followed by robust pose optimization [4,5,17,18,19]. Other formulations explore relative pose regression (RPR) across image pairs or short sequences [6]. Despite their efficiency and simplicity, these approaches remain sensitive to dataset artifacts such as uneven viewpoint distributions and orientation bias, which can hinder generalization.

To alleviate such issues, recent work has investigated synthetic-view augmentation. LENS [7] generates spatially balanced virtual viewpoints using NVS and mixes them with real observations for localization training, reporting consistent improvements on benchmarks such as Cambridge Landmarks dataset  [1]. While LENS demonstrated the potential of NVS for localization, it relies on uniform grid sampling, which lacks explicit reasoning about scene geometry and visibility. This limitation becomes critical in unordered collections where camera poses are irregular. LEGS advances this by introducing a visibility-aware SE(3) selection strategy that anchors virtual viewpoints to the underlying SfM graph, ensuring both geometric validity and triangulation strength. Subsequent studies have extended this idea through differentiable rendering and geometry-consistent synthesis, demonstrating that NVS-based augmentation can bridge data gaps and improve localization robustness.

We build on this data-centric perspective but differ in two key aspects: (i) instead of uniform grid placement, we introduce a pose proposal-and-selection strategy in SE(3) that accounts for visibility and baseline geometry to promote both diversity and triangulation strength; and (ii) we employ 3DGS [9] as a fast, scalable renderer compatible with existing localization pipelines. This results in a trajectory-agnostic, geometry-consistent augmentation policy suitable for unordered or co-located image collections.

### 2.3. Novel View Synthesis

NVS has recently become a powerful tool for creating viewpoint-diverse data, verifying pose consistency, and testing robustness under varying conditions. Neural radiance field (NeRF) models [20] have enabled high-quality photorealistic synthesis from posed images. Subsequent variants improved flexibility and realism in more complex settings: NeRF-W [21] handles unconstrained photo collections, mip-NeRF 360 [22] addresses aliasing and unbounded scenes, and Zip-NeRF [23] introduces anti-aliased grid-based representations. These developments have made NVS practical for localization research, allowing synthetic views to augment datasets, test geometric consistency, and verify pose estimates through rendering.

More recently, 3DGS [9] reformulated scene representations using explicit Gaussian primitives and visibility-aware rasterization, enabling real-time rendering and much faster training. This efficiency is essential for scaling up data synthesis across large scenes. Follow-up work such as Mip-Splatting [24] further improves anti-aliasing and stability across different scales and focal lengths—an important factor when sampling diverse baselines without visual artifacts. In practice, many pipelines still initialize from SfM camera poses and sparse 3D structures, then employ either NeRF-based or 3DGS-based synthesis. Therefore, the problem of where to place virtual cameras and how to balance visibility and baseline geometry becomes just as crucial as the rendering method itself.

Our approach operates precisely at this intersection: it retains the geometric prior from SfM, introduces a visibility- and baseline-aware camera selection strategy, and employs 3DGS to make large-scale synthetic augmentation both practical and efficient.

## 3. Methodology

Algorithm 1 summarizes the overall LEGS framework. Our LEGS framework comprises two main stages. In Stage 1, we perform SfM to recover camera poses and sparse geometry, followed by a sanitization step that removes mirrored or ghost structures, yielding a clean and consistent 3D model. A vanilla 3DGS proxy is then trained on this sanitized SfM output to provide a fast, visibility-aware scene representation. In Stage 2, we introduce our core contribution—a visibility- and baseline-aware pose proposal and selection strategy that operates in full SE(3) space. Using the 3DGS proxy, LEGS generates and filters virtual camera candidates to ensure physically valid, diverse viewpoints before rendering high-fidelity synthetic images. Together, these two stages form a geometry-consistent and scalable pipeline for robust synthetic-view-based visual localization.

### 3.1. Sanitized SfM and Vanilla 3DGS

Visual localization requires labeled pairs of images and corresponding camera poses $\left\{\left(I_{i},T_{i}\right)\right\}$ with $T_{i}\in \mathrm{SE}\left(3\right)$. While some datasets provide accurate ground-truth poses from auxiliary sensors such as LiDAR, stereo, or depth cameras, most practical sources rely solely on RGB imagery: (i) monocular sequences captured along continuous trajectories, (ii) unordered image collections acquired from arbitrary viewpoints without temporal continuity, and (iii) large-scale Internet photo sets collected from heterogeneous devices. In these settings, SfM is the standard mechanism for estimating camera poses and a sparse 3D structure, and many benchmarks (e.g., Cambridge Landmarks [1]) are distributed with, or can be reconstructed into, COLMAP-compatible models whose text/binary outputs are widely used in downstream toolchains.

RGB-only reconstructions, however, are vulnerable to pose ambiguities and place-recognition errors that yield duplicated or spurious geometry—often termed ghost or doppelgänger structures. Specifically, ghost geometry refers to floating, semi-transparent artifacts without physical correspondence, typically appearing when the optimization compensates for misaligned poses. Doppelgänger structures refer to spatially misaligned duplicates of a single physical entity. This phenomenon arises when repetitive or symmetrical patterns cause feature-matching ambiguities, leading to erroneous triangulation. Consequently, a single real-world object is incorrectly reconstructed as multiple redundant structures at different spatial coordinates, severely degrading the reliability of the geometric proxy.
**Algorithm 1** LEGS Framework**Require:**  1: $I={\left\{\left(I_{i},C_{i},R_{i},K_{i}\right)\right\}}_{i=1}^{n}$            ▹ Input images with 6-DoF poses and intrinsics  2: ${\tilde{P}}_{\mathrm{GS}}$                        ▹ Geometric proxy optimized via 3DGS  3: thresholds $\left\{t,\tau_{\mathrm{fov}},\theta_{\mathrm{elev}},\tau_{\mathrm{occ}},r_{\min},\tau_{\mathrm{nms}}\right\}$             ▹ Hyperparameters for selection**Ensure:** Synthetic view set $D_{\mathrm{syn}}$ and virtual camera set V  4: $B\;\leftarrow \;\mathrm{AABB}(\left\{C_{i}\right\})$; dilate 10%           ▹ Define scene boundary with safety margin  5: $L\;\leftarrow \;∥b_{\max}-b_{\min}{∥}_{2}$; spacing d←0.05L; build grid cells ν              ▹ Discretize space  6: Initialize $V,D_{\mathrm{syn}}\;\leftarrow \;Ø$
  7: VTX←GridVertices(B,d)                ▹ Generate potential grid-based viewpoints  8: **for** each vertex $v_{j}\in \mathrm{VTX}$ **do**              ▹ Stage 1: Grid-based viewpoint proposal  9:     $i^{⋆}\;\leftarrow \;\arg\min_{i}{∥v_{j}-C_{i}∥}_{2}$; $\left(C,R,K\right)\;\leftarrow \;\left(v_{j},R_{i^{⋆}},K_{i^{⋆}}\right)$10:     **if** $\mathrm{dist}\left(C,{\tilde{P}}_{\mathrm{GS}}\right)\leq r_{\min}$ or forward-cone collision **then continue**       ▹ Prune internal views11:     **end if**12:     V←V∪{(K,R,C)}13: **end for**14: **for** each cell ν **do**               ▹ Stage 2: Pairwise interpolation for density15:     **for** each unordered pair $\left(i,j\right)\in I_{\nu }$ **do**16:         Compute ${\mathrm{overlap}}_{\mathrm{geom}}\left(i,j\right)$;17:         **if** ${\mathrm{overlap}}_{\mathrm{geom}}<\tau_{\mathrm{fov}}$ **then continue**18:         **end if**                            ▹ Check FOV connectivity19:         Compute ${\mathrm{overlap}}_{\mathrm{feat}}\left(i,j\right)$;20:         **if** ${\mathrm{overlap}}_{\mathrm{feat}}>1-\tau_{\mathrm{fov}}$ **then continue**21:         **end if**                          ▹ Ensure enough parallax22:         **for** k=1 to *t* **do**23:            u←k/(t+1); interpolate C,R via slerp; apply jitter           ▹ Populate gaps24:            **if** $|\mathrm{elev}\left(R\right)|\;>\;\theta_{\mathrm{elev}}$ or $\mathrm{occ}\left(C,R\right)\;<\;\tau_{\mathrm{occ}}$ **then continue**25:            **end if**                                ▹ Filtering26:            Harmonize $f_{j}=\mathrm{median}\left\{f_{x},f_{y}\right\}$; choose $K_{ij}$27:            $V\;\leftarrow \;V\cup \{\left(K_{ij},R,C\right)\}$28:         **end for**29:     **end for**30: **end for**31: Apply NMS in SE(3) space with threshold $\tau_{\mathrm{nms}}$     ▹ Prune redundant poses for diversity32: **for** each (K,R,C)∈V **do**33:     Render via 3DGS ⇒ image $\in D_{\mathrm{syn}}$           ▹ High-throughput image synthesis34: **end for**35: Train localization model on $D_{\mathrm{real}}\cup D_{\mathrm{syn}}$          ▹ Final data-augmented training

To mitigate such artifacts before any NVS, we introduce a dedicated sanitization stage inspired by learned disambiguation methods such as Doppelgangers [25] and Doppelgangers++ [26]. In our framework, we specifically implement the sanitization and pose refinement pipeline proposed in [27], which leverages 3DGS as a high-fidelity geometric proxy to detect and correct spatial misalignments. When initial image poses are available, we adopt them as provided; otherwise, we reconstruct the camera triplets $\left(K_{i},R_{i},C_{i}\right)$ via incremental SfM. Following the methodology in [27], we then perform a 3DGS-based re-alignment: ambiguous or inconsistent image pairs are identified through rendering-based verification, and suspect camera poses are iteratively refined by minimizing the photometric and geometric discrepancy between the real captures and the 3DGS-rendered proxies. This process effectively prunes doppelgänger structures and yields a sanitized SfM backbone that is highly reliable for subsequent viewpoint synthesis.

**Figure 1:**
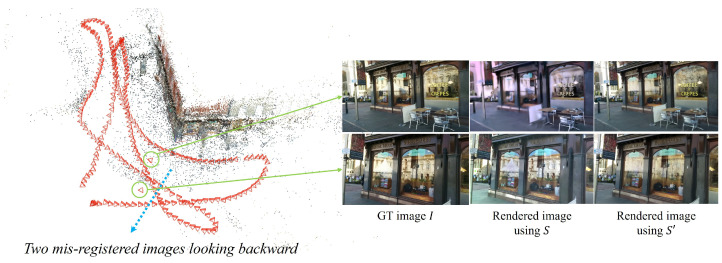
Visualization of the *Shop Facade* scene from the Cambridge Landmarks dataset. The original SfM reconstruction contains two misregistered camera poses that lead to visible artifacts and degraded rendering quality when synthesized using 3DGS. To ensure stable and geometry-consistent view generation, these erroneous poses must be corrected or removed through a doppelganger-free sanitization process before training the 3DGS proxy and applying the proposed LEGS framework.

Flagged subsets are refined with constrained bundle adjustment and visibility checks, producing a cleaned pose graph and a more reliable sparse model for subsequent rendering and training. For example, in the *Shop Facade* scene of the Cambridge Landmarks dataset (Figure 1), which is composed of images extracted from a video sequence, most cameras consistently face the main facade while two images are oriented in the opposite direction, clearly separated in position from the others. Such inconsistent poses are implausible in the real scene and indicate misaligned or misregistered images caused by the inherent limitations of SfM. When a dataset contains many such errors, the reconstructed model exhibits duplicated or mirrored geometry—commonly referred to as ghost or doppelgänger structures. To mitigate these artifacts, learned disambiguation techniques such as Doppelgangers and Doppelgangers++ are essential prior to downstream localization or view-synthesis stages.

From the sanitized SfM model, we train a vanilla 3DGS representation [9] to serve as a fast, visibility-aware proxy for the scene. We intentionally avoid newer 3DGS variants and keep the SfM poses fixed to isolate the effects of our pose generation and selection framework. This proxy provides two essential capabilities for the later stages: (i) efficient per-view visibility queries and (ii) high-throughput rendering of candidate virtual views.

Given $\left\{K_{i},R_{i},C_{i}\right\}$ and $P_{\mathrm{sparse}}$, we optimize a 3DGS scene $G={\left\{g_{k}\right\}}_{k=1}^{K}$ by minimizing a photometric reconstruction loss with regularization on opacity and covariance. Differentiable splatting replaces the expensive volumetric integration used in radiance-field models, yielding fast convergence and high rendering efficiency. After convergence, we extract the Gaussian centers $P_{\mathrm{GS}}=\left\{x_{k}\right\}$ as a dense geometric proxy; typically $|P_{\mathrm{GS}}\left|\;\gg \;\right|P_{\mathrm{sparse}}|$, which substantially improves co-visibility reasoning and coverage of fine details.

To ensure stable and efficient geometric queries, we apply three post-processing steps: (i) Poisson-disk subsampling to obtain ${\tilde{P}}_{\mathrm{GS}}$ with well-spaced points; (ii) DBSCAN-based removal of small or isolated clusters to eliminate transient or floating artifacts; and (iii) DBSCAN-based removal of small or isolated clusters. The clustering parameters are adaptively set to ensure scale-invariance: the neighborhood radius is defined as ϵ=0.02L, and the minimum cluster size is min_samples=0.001N, where *N* denotes the total number of sparse SfM points. This step effectively prunes isolated ghost geometry that lacks sufficient spatial density to represent physical structures, thereby ensuring that the 3DGS proxy ${\tilde{P}}_{GS}$ is initialized from a reliable geometric foundation.

The choice of 3DGS as our rendering engine provides a fundamental shift in efficiency and geometric utility compared to previous radiance-field-based approaches like LENS [7]. While NeRF-based models [20] achieve high photorealism, their reliance on expensive volumetric sampling limits their throughput for large-scale data augmentation. In contrast, 3DGS reformulates the scene as explicit Gaussian primitives, enabling real-time rasterization and significantly higher synthesis throughput within practical budgets. Beyond speed, the explicit nature of the 3DGS proxy ${\tilde{P}}_{GS}$ facilitates fast, visibility-aware occupancy checks that are critical for our SE(3)-aware pose selection, a capability that implicit representations lack. Consequently, LEGS is uniquely positioned to handle the high-volume synthesis required for robust localization in complex, unordered environments.

In summary, Stage 1 yields a sanitized, geometry-consistent 3DGS proxy that underpins our subsequent camera pose proposal, visibility-aware selection, and large-scale synthetic-view generation.

### 3.2. LEGS: Detailed Pipeline Description

Below we explain each stage in detail, using the notation introduced in Section 3.1 for the 3DGS-derived proxy ${\tilde{P}}_{GS}$ and the SfM camera triplets $\left(K_{i},R_{i},C_{i}\right)$. Here, $K_{i}$ denotes the intrinsic matrix representing focal length and principal points, $R_{i}$ signifies the rotation matrix, and $C_{i}$ represents the camera center in 3D space.

We first compute a single global axis-aligned bounding box (AABB) $B=[b_{min},b_{max}]$ that encompasses all real camera centers $\left\{C_{i}\right\}$. This global AABB is then expanded by 10% along each axis to allow moderate extrapolation beyond the observed region. The scene scale is defined as $L=\;∥b_{\max}-b_{\min}{∥}_{2}$. We then divide B into a regular 3D grid with voxel size d=0.05L. This discretization localizes computations and confines subsequent pair enumerations to cell-level neighborhoods. This discretization localizes computations by confining subsequent pair enumerations to cell-level neighborhoods. We define $I_{v}=\left\{i|C_{i}\in v\right\}$ as the set of indices of real cameras whose centers fall within grid cell *v*. Consequently, the naive $O(n^{2})$ complexity of camera pair comparisons, where *n* is the total number of cameras, is reduced to $\sum_{v}O(|I_{v}{|}^{2})$. In large-scale scenes, the condition $|I_{v}|\;\ll \;n$ typically holds because the voxel size d=0.05L is small relative to the total scene volume, ensuring that only a local subset of cameras is compared. For smaller datasets where this disparity is less pronounced, the absolute computational cost remains low, and the grid still serves to preserve geometric locality.

The choice of these specific values—including the 10% AABB expansion, the voxel size d=0.05L, and the geometric overlap constraints—was determined empirically through preliminary tests. These parameters were selected to maximize the diversity of synthesized viewpoints while ensuring that the computational complexity remains scalable for large-scale scenes.

To obtain an initial set of uniformly distributed virtual camera candidates, we adopt a LENS-style vertex seeding strategy [7]. Candidate cameras are placed at grid vertices VTX=GridVertices(B,d). For each vertex $v_{j}$, we identify the nearest real camera

$$i^{⋆}=\arg\min_{i}{∥v_{j}-C_{i}∥}_{2},$$

and inherit its orientation ($R_{i^{⋆}}$) and intrinsics ($K_{i^{⋆}}$):

$$\left(C_{j},R_{j},K_{j}\right)=\left(v_{j},R_{i^{⋆}},K_{i^{⋆}}\right).$$

To avoid physically implausible viewpoints, we apply a safety filter using the 3DGS proxy ${\tilde{P}}_{\mathrm{GS}}$: any vertex with $\mathrm{dist}\left(C_{j},{\tilde{P}}_{\mathrm{GS}}\right)\leq r_{\min}$ is removed. The surviving candidates provide a uniform, topology-aware initialization that retains local viewing characteristics from the nearest real cameras. This grid-based seeding strategy works particularly well for trajectory-like datasets where images are extracted from continuous video frames, as neighboring frames naturally exhibit smooth spatial transitions. However, for unordered or irregularly spaced image collections, such uniform grids alone may fail to capture the true viewpoint diversity, necessitating additional SE(3)-aware sampling and selection as introduced in our framework.

To create meaningful baselines for interpolation, we consider only cameras within the same grid cell. For each cell ν, we gather $I_{\nu }=\left\{i|C_{i}\in \nu \right\}$ and form unordered camera pairs (i,j). A two-stage filtering process eliminates redundant or invalid pairs. First, we estimate coarse geometric overlap from camera orientations and fields of view:

$${\mathrm{overlap}}_{\mathrm{geom}}\left(i,j\right)=1-\frac{\theta_{ij}}{\frac{1}{2}\left(\phi_{i}+\phi_{j}\right)},$$

where $\theta_{ij}$ is the angular difference between orientations and $\phi_{i},\phi_{j}$ are the horizontal FoVs. Pairs with ${\mathrm{overlap}}_{\mathrm{geom}}<0.5$ are discarded. To avoid degenerate cases where two cameras observe nearly identical regions, pairs with ${\mathrm{overlap}}_{\mathrm{geom}}>0.8$ are also removed, as excessively overlapping views contribute little new geometric information. This first-stage filtering relies solely on FoV geometry and is computationally efficient, allowing rapid pruning of unlikely pairs before any feature-level operations.

For the remaining pairs, a refined overlap ratio

$${\mathrm{overlap}}_{\mathrm{feat}}\left(i,j\right)=\frac{|M_{ij}|}{\min(|F_{i}|,|F_{j}|)},$$

is computed from SfM feature correspondences $M_{ij}$. This second-stage check measures the true visual overlap in feature space, providing a more reliable indicator of mutual visibility. Pairs with ${\mathrm{overlap}}_{\mathrm{feat}}>1-\epsilon_{\mathrm{fov}}$ are pruned, ensuring that only moderately overlapping, geometrically complementary neighbors are preserved. This hierarchical screening—coarse FoV-based filtering followed by fine feature-based validation—balances computational efficiency and accuracy while suppressing both insufficient and redundant baselines.

For each retained pair (i,j), we interpolate *t* intermediate viewpoints to increase sampling density. Each virtual camera center is linearly interpolated as $C=\left(1-u\right)C_{i}+uC_{j}$, and orientation is blended on SO(3) using spherical interpolation $q=\mathrm{slerp}(q_{i},q_{j};u)$ followed by R=R(q). A small random perturbation, ApplyJitter, decorrelates adjacent poses and avoids degenerate baselines. To maintain realistic perspectives, we reject views whose elevation angle exceeds a predefined limit $|\mathrm{elev}\left(R\right)|>\theta_{\mathrm{elev}}$.

Each interpolated pose is verified against the 3DGS proxy to remove physically invalid viewpoints. First, cameras that are too close to the surface are filtered out using the same minimum-distance rule as in the seeding stage, since such poses may lie inside walls or objects and lead to rendering artifacts. Second, we check whether each camera is directly facing an obstacle through a *front-aware collision check* along its optical axis occ(C,R):

$$\min_{x\in {\tilde{P}}_{\mathrm{GS}}}\left\{{∥x-C∥}_{2}\;|\;∠\;\left(\frac{x-C}{∥x-C∥},R^{⊤}e_{z}\right)\leq \phi \right\}>r_{\min},$$

where ϕ is a small cone half-angle. If any geometry lies too close in front of the camera (closer than $r_{\min}$ within this cone), the pose is considered invalid and removed.

To maintain consistent fields of view across heterogeneous cameras, we harmonize intrinsics before accepting new poses. If local neighbors exhibit large focal discrepancies, we compute a median focal length

$$f_{j}=\mathrm{median}\left\{f_{x}\left(i\right),f_{y}\left(i\right)|i\;\in \;N_{k}\left(C\right)\right\},\;K_{j}\leftarrow \mathrm{ClampFoV}\left(K_{i^{⋆}},f_{j}\right),$$

where $N_{k}\left(C\right)$ denotes the *k* nearest real cameras. If $K_{i}$ and $K_{j}$ are similar, we reuse $K_{i}$; otherwise, we adopt the harmonized $K_{j}$. Each accepted configuration $\left(K_{ij},R,C\right)$ is appended to V. Since interpolation is localized within cells and *t* is small, the number of valid virtual poses scales predictably with cell density.

After proxy validation, redundant poses may still remain. We therefore apply non-maximum suppression (NMS) in SE(3) space to retain only diverse, representative viewpoints. For two poses $\left(R_{1},C_{1}\right)$ and $\left(R_{2},C_{2}\right)$, the distance metric is defined as

$$d_{\mathrm{SE}(3)}\left(\left(R_{1},C_{1}\right),\left(R_{2},C_{2}\right)\right)=\sqrt{\frac{∥C_{1}-C_{2}{∥}_{2}^{2}}{\sigma_{t}^{2}}+\frac{\theta {\left(R_{1}^{⊤}R_{2}\right)}^{2}}{\sigma_{r}^{2}}},$$

where $\sigma_{t}$ and $\sigma_{r}$ control the sensitivity to translation and rotation differences. Using this metric, we measure how close two viewpoints are in both position and orientation. Poses that fall within a small neighborhood ($d_{\mathrm{SE}(3)}<\tau_{\mathrm{nms}}$) are considered redundant, and only one representative pose is retained. Poses that fall within a small neighborhood are considered redundant, and only one representative pose is retained. In our implementation, this selection is performed through a greedy pruning process: the first candidate pose in the proposed set that satisfies all prior visibility and collision checks is accepted as the representative, and all other candidates within the distance threshold $\tau_{nms}$ are suppressed. This process effectively eliminates densely clustered or nearly identical views, producing a blue-noise-like spatial distribution that balances coverage and diversity across the scene. This process eliminates densely clustered or nearly identical views, producing a blue-noise-like spatial distribution that balances coverage and diversity across the scene.

Finally, each accepted pose (K,R,C) is rendered by the trained 3DGS model, producing high-fidelity RGB images at real-time throughput. The resulting synthetic dataset $D_{\mathrm{syn}}$ augments the real dataset $D_{\mathrm{real}}$ for joint training of the localization network.

In summary, LEGS integrates grid-based vertex seeding, cell-local interpolation, and proxy-guided geometric filtering into a computationally predictable framework that generates geometrically valid and photometrically stable virtual views, serving as high-quality synthetic data for downstream visual localization and novel-view synthesis tasks.

## 4. Experimentation

We validated the proposed LEGS framework on two datasets of different characteristics: the publicly available Cambridge Landmarks dataset [1], and an in-house dataset collected specifically for this study.

### 4.1. Implementation Details

All experiments, including 3DGS optimization, synthetic view generation, and localization model training, were conducted on a workstation equipped with a single NVIDIA RTX A5000 GPU (NVIDIA Corporation, Santa Clara, CA, USA). The memory capacity of the A5000 was sufficient to handle the 3DGS training and the subsequent high-throughput image synthesis for all evaluated datasets, including the large-scale Cambridge Landmarks and our in-house scenes. On average, 3DGS optimization for a single scene was completed within practical timeframes, and the rendering process maintained real-time throughput, facilitating the generation of thousands of augmented views without significant computational bottlenecks. For 3DGS optimization, we followed a standard schedule with 30,000 iterations to ensure stable geometric convergence. During the viewpoint proposal stage, candidates were sampled within an elevation range of $\theta_{elev}=\pm 15^{\circ }$. To ensure the quality of the synthesized images, we applied multiple selection constraints: a minimum field-of-view overlap $\tau_{fov}=0.6$, an occlusion threshold $\tau_{occ}=0.3$ to filter out low-confidence renderings, and a minimum camera-to-geometry distance $r_{\min}=0.1\;m$ to avoid near-wall artifacts. Furthermore, geometric and feature-based overlap thresholds were set to $\tau_{geom}=0.5$ and $\tau_{feat}=0.8$ to balance pose diversity and visibility. Finally, to prune redundant candidates, we applied SE(3)-aware NMS with a distance threshold $\tau_{nms}=1.0$, where the sensitivity parameters for translation and rotation were set to $\sigma_{t}=0.2\;m$ and $\sigma_{r}=15{}^{\circ}$, respectively.

### 4.2. Cambridge Landmarks Dataset

The Cambridge Landmarks dataset [1] is a well-established benchmark for evaluating visual localization performance. It was originally reconstructed using VisualSfM [28] from handheld smartphone videos captured in outdoor urban environments. The dataset comprises six landmark scenes in total; however, only four of them are commonly used for visual localization research due to their higher reconstruction quality and more stable camera pose annotations. In line with prior work, we also used these four scenes in our experiments. Each selected scene provides SfM-derived ground-truth camera poses and 3D structure, enabling a consistent evaluation of pose regression methods under realistic outdoor conditions.

**Figure 2:**
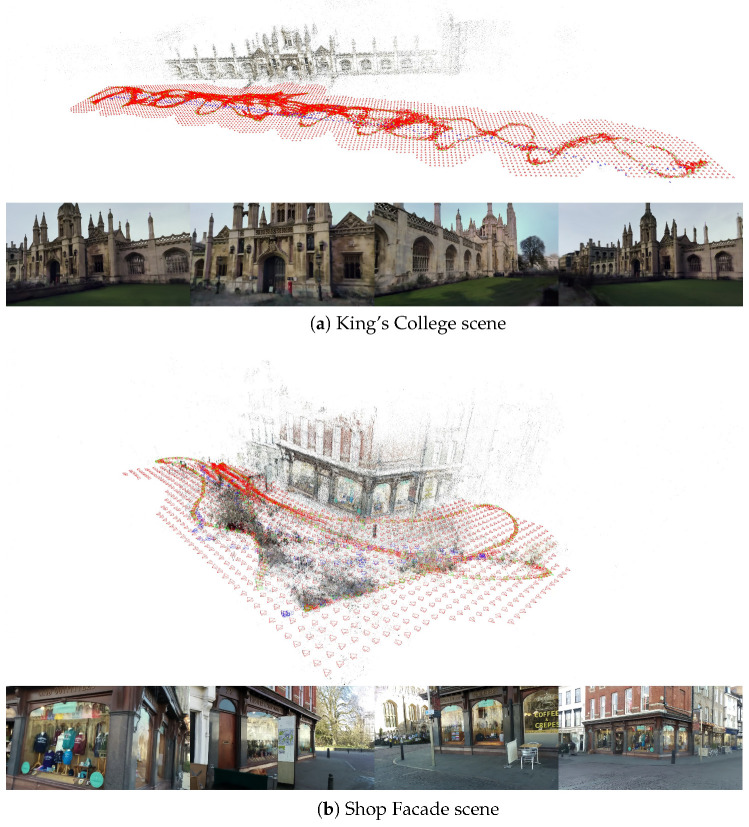

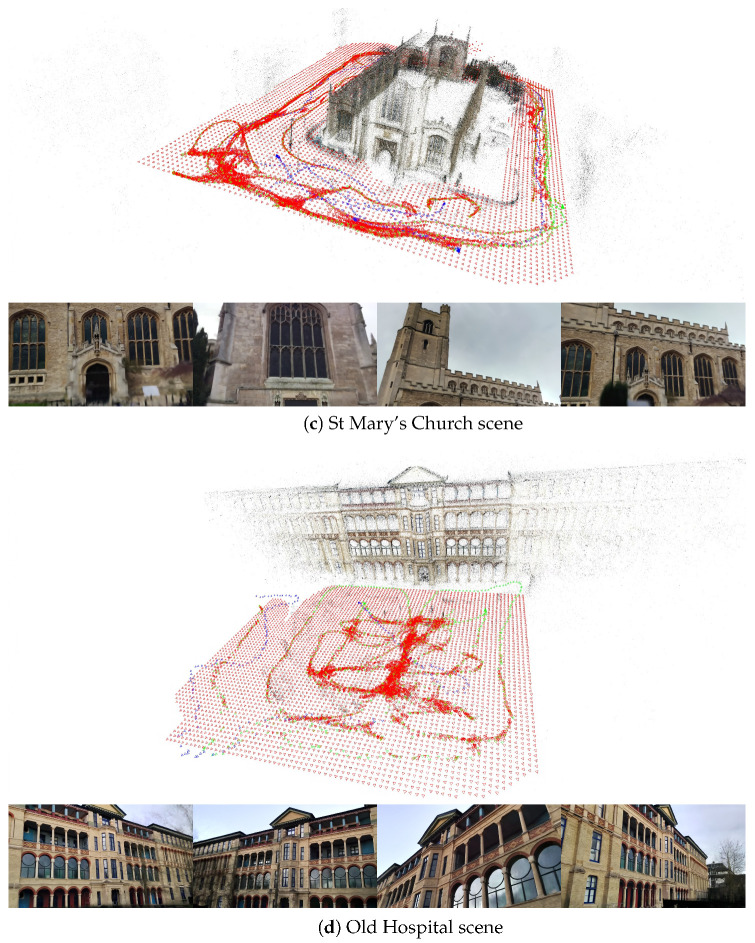
Visualization of the Cambridge Landmarks dataset used for visual localization. (**a**) *King’s College*, (**b**) *Shop Facade*, (**c**) *St Mary’s Church*, and (**d**) *Old Hospital*. **Green:** camera poses used for training visual localization. **Blue:** camera poses reserved for testing. **Red:** virtual camera poses generated by our LEGS framework. Sample images rendered using 3DGS are also shown.

Because each image was extracted from a continuous video sequence, the camera centers and orientations are spatially and directionally coherent, forming nearly sequential trajectories rather than randomly distributed viewpoints. As illustrated in Figure 2, the camera poses follow smooth motion paths with gradual viewpoint changes, reflecting the natural movement of the handheld device during video capture. This sequential acquisition pattern contributes to strong spatial redundancy, which can be advantageous for SfM reconstruction but may also introduce viewpoint bias in pose regression tasks.

This issue is particularly critical for our framework, since erroneous poses directly affect the generation of virtual cameras. If these outlier poses are not corrected, the subsequent interpolation and viewpoint sampling stages may inherit their geometric errors, producing physically implausible synthetic views. Therefore, sanitizing the SfM model before view generation is a key prerequisite for ensuring reliable camera proposals and high-quality novel view synthesis.

As illustrated in Figure 1, even a small number of misaligned cameras can distort the spatial structure of the scene and lead to incorrect visibility reasoning. By applying a doppelganger-aware correction procedure prior to virtual-camera generation, LEGS automatically detects and re-aligns these inconsistent poses, restoring geometric coherence across the model and preventing ghost artifacts from propagating into the synthesized views.

We evaluated our LEGS framework using a standard SCR framework, SeqACE [5], which provides a strong baseline for assessing visual localization accuracy. As shown in Table 1, applying our pipeline led to consistent improvements in median localization accuracy across the evaluated scenes. Following standard evaluation protocols in visual localization, we report the median translation and rotation errors. While DSAC* exhibits superior absolute accuracy in the table, it is important to note the trade-off between precision and practical efficiency. DSAC* typically requires extensive training time and larger storage for scene-specific maps. In contrast, we adopt SeqACE as our baseline due to its high efficiency in both training and inference, making it more suitable for real-time applications. The results demonstrate that LEGS effectively bridges the accuracy gap between such efficient encoders and more computationally intensive models. By providing geometry-consistent synthetic augmentation, LEGS allows practical models to achieve performance levels closer to state-of-the-art heavy baselines without compromising their architectural efficiency.

For the *Shop Facade* scene, the corrected SfM model demonstrated more stable training behavior and slightly lower localization error compared to the uncorrected SfM baseline. Although the performance gain is moderate rather than dramatic, it highlights the importance of reliable geometric consistency when using SfM-derived poses as supervision for learning-based localization.

The improvement can be attributed to two main factors. First, sanitizing the SfM model removes a small number of misaligned poses that would otherwise propagate spatial inconsistencies to downstream training, resulting in cleaner and more coherent supervision signals. Second, by refining or discarding these erroneous poses, the resulting virtual cameras generated through our LEGS pipeline are distributed more uniformly and remain geometrically plausible, which leads to more stable optimization during pose regression.

It is worth noting, however, that the Cambridge Landmarks dataset was created from continuous handheld video sequences, where camera centers and orientations are already densely and sequentially sampled. As illustrated in Figure 2, adjacent frames often capture nearly identical viewpoints, leaving limited room for augmentation through interpolated virtual cameras. Consequently, our pose proposal-and-selection mechanism yields only incremental improvements in this dataset, since the available real frames already provide strong spatial continuity and dense coverage. Nevertheless, the results confirm that even in such video-based scenarios, removing outlier poses and enforcing geometric consistency still contributes measurable gains in localization performance.

Overall, these findings demonstrate that our method enhances the robustness and reliability of visual localization pipelines built upon SfM reconstructions. By ensuring that the training data are free from duplicated geometry and misaligned poses, LEGS provides a stable foundation for learning-based localization, yielding improved accuracy and smoother convergence without the need for additional supervision or manual filtering.

### 4.3. In-House Dataset

To further evaluate the generality and robustness of LEGS, we constructed an in-house dataset captured in diverse real-world environments, including indoor scenes. All images were taken using a Samsung Galaxy S20+ smartphone (Samsung Electronics, Republic of Korea) under consistent illumination and static conditions to minimize motion blur and appearance variations. The dataset comprises four scenes—*Show Room*, *Testbed*, *Conference Room*, *Stairs*—each containing several hundred high-resolution photographs captured from various viewpoints. Detailed statistics for each scene in the in-house dataset are provided in Table 2.

Unlike the Cambridge Landmarks dataset, where frames are sequentially extracted from continuous video streams, our in-house dataset consists of unordered, independently captured images. Each scene includes photographs taken from irregular positions and viewing directions, without any fixed trajectory or temporal order. This configuration reflects a more realistic but challenging scenario for SfM reconstruction, as geometric overlap between views varies greatly and feature correspondence becomes less reliable.

**Figure 3:**
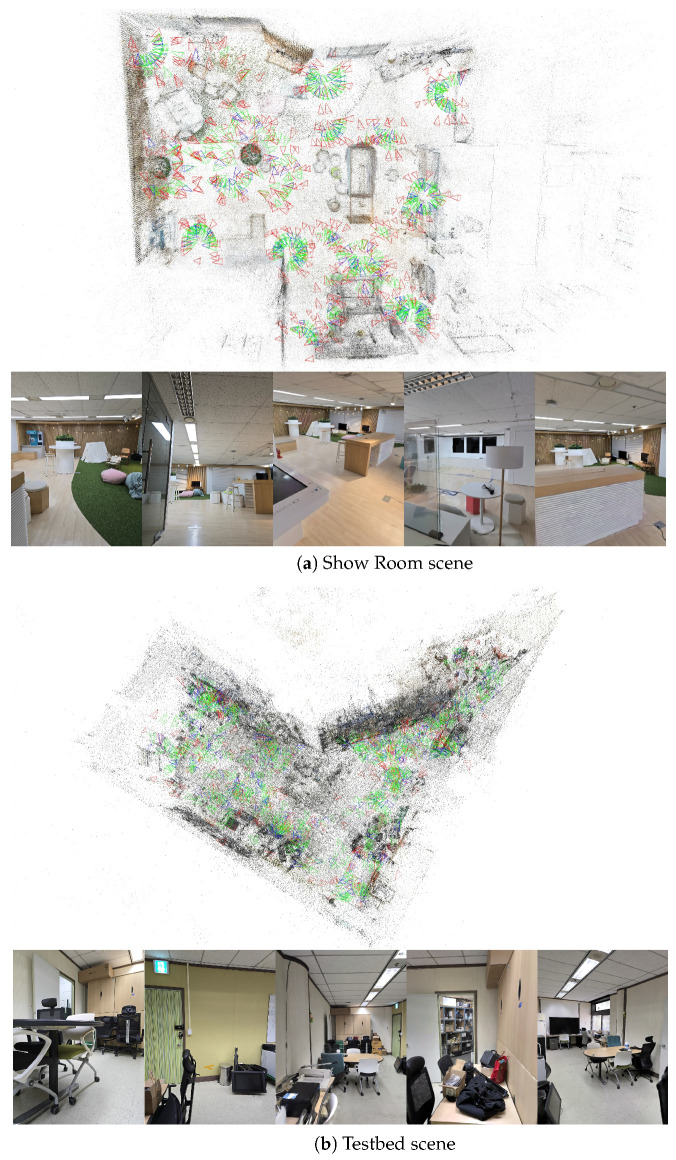

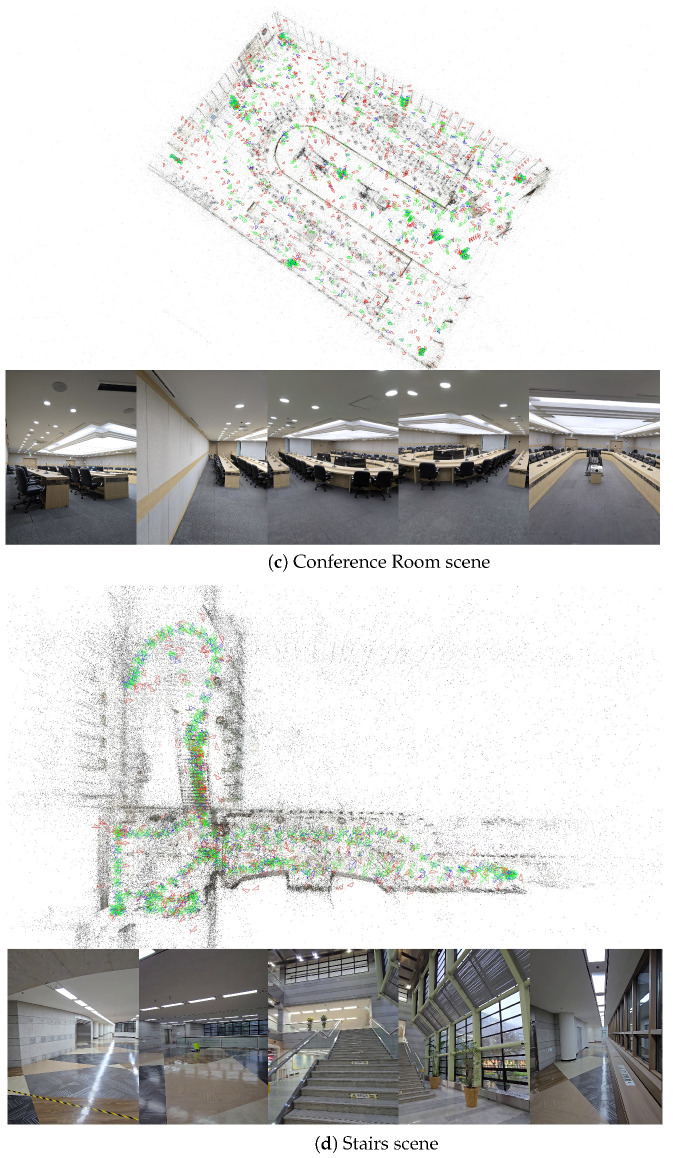
Examples from our in-house dataset with corresponding images rendered using 3DGS. **Green:** camera poses used for training visual localization. **Blue:** camera poses reserved for localization testing. **Red:** virtual camera poses generated by our LEGS framework. Due to the large number of proposed virtual cameras, only a sampled subset is visualized for clarity.

Under such conditions, conventional SfM pipelines often produce misaligned poses or duplicated geometry when repetitive patterns, reflective surfaces, or insufficient parallax are present. Our SfM sanitization procedure mitigates these issues by detecting and correcting erroneous poses before 3DGS training. The resulting scenes reconstructed through the LEGS pipeline are visualized in Figure 3, showing clean geometry and stable novel view synthesis, while the corresponding visual localization performance is summarized in Table 3.

It should be noted that the evaluation on the in-house dataset is specifically designed to measure the relative improvement provided by the LEGS augmentation framework, rather than to perform a cross-architectural benchmark of various localizers. While comprehensive comparisons between models such as DSAC* and SeqACE are provided for the Cambridge Landmarks dataset, the in-house experiments focus on isolating the impact of our SE(3)-aware pose selection strategy in unordered capture scenarios. By maintaining a fixed localizer backbone, we demonstrate that the observed gains are a direct consequence of the enhanced data distribution provided by LEGS. As LEGS operates at the data level by improving the geometric coverage of the training set, its benefits are fundamentally model-agnostic.

To evaluate the specific impact of our sampling strategy, we compare the proposed method against a ‘Grid-only’ baseline, which serves as a functional proxy for the LENS [7] methodology. Since LENS is primarily characterized by uniform grid-based sampling for view synthesis, our ‘Grid-only’ implementation adopts this exact logic while utilizing the same 3DGS rendering engine. While the original LENS utilizes NeRF-based synthesis, we adopted the 3DGS engine for our implementation of this baseline to ensure that the evaluation focuses on the pose-selection logic rather than renderer performance. This setup allows us to isolate the geometric benefits of SE(3)-aware pose selection from the rendering performance, providing a controlled data-centric ablation study. Together, these results demonstrate the effectiveness of the proposed framework in unordered, real-world capture scenarios.

While grid-based sampling strategies such as LENS [7] are generally sufficient for datasets like Cambridge Landmarks and the *Stairs* scene of our in-house dataset, where viewpoints are captured along smooth and continuous trajectories, they face clear limitations when multiple images share almost identical positions but differ significantly in orientation like our in-house dataset. In such cases, uniformly placing virtual cameras on a 3D grid may result in redundant or even missing samples, since several real viewpoints can collapse into the same grid cell. For instance, when two images are captured from nearly the same location but with different headings, a grid-only method may generate a single synthetic view aligned with the grid vertex, thereby failing to represent the directional diversity present in the scene.

This limitation becomes far more evident in our in-house dataset, where many images are taken from similar spatial locations but with different orientations toward surrounding objects. Here, angular diversity plays a much greater role than positional variation, and generating virtual cameras that account for these orientation differences is essential for achieving balanced coverage of the visual field. Our LEGS framework explicitly addresses this issue by operating in the full SE(3) space—jointly considering both translation and rotation—to propose and select virtual cameras that capture complementary viewpoints. This visibility- and baseline-aware selection mechanism prevents angular redundancy and ensures that generated virtual views reflect the true distribution of orientations within the scene.

As a result, the in-house dataset demonstrates a notably larger performance gain than Cambridge Landmarks. By effectively synthesizing viewpoint-diverse virtual cameras, LEGS enhances geometric coverage and feature overlap, leading to more consistent SfM alignment and improved downstream visual localization accuracy. These findings confirm that the proposed approach is particularly advantageous in unconstrained capture environments where spatial overlap is high but orientation variation dominates, a setting that closely reflects real-world data acquisition scenarios.

### 4.4. Ablation Study

#### 4.4.1. Impact of Augmented View Density

Table 4 summarizes the sensitivity of visual localization performance to the number of synthesized augmented images $n_{v}$. Our evaluation reveals a clear correlation between the density of the augmented training set and the resulting pose accuracy. When an insufficient number of synthetic views is provided, the localizer fails to fully bridge the geometric gaps in the sparse capture regions, leading to higher median errors. Increasing the volume of synthetic data to $n_{v}$ yields a substantial improvement across all scenes, confirming that denser SE(3) coverage effectively densifies the training signal. However, we observe a saturation point at $2n_{v}$, where further increasing the image count provides diminishing returns in accuracy. This suggests that once the fundamental geometric distribution of the scene is sufficiently represented, redundant viewpoints offer limited additional information for the image encoder.

Regarding computational efficiency, a granular time comparison for different values of $n_{v}$ during the rendering stage is omitted, as its impact on the overall pipeline is of limited practical significance. Given the high-throughput nature of the 3DGS framework, synthesizing several hundred additional images results in a time difference that is negligible when compared to the durations of the SfM and 3DGS optimization stages. For instance, while SfM and 3DGS optimization can take up to several hours depending on scene scale, the rendering of $n_{v}$ augmented views is completed in mere seconds or minutes. Instead, the primary influence of $n_{v}$ is reflected in the localization model’s training phase, where the increased data volume naturally extends the training duration. A detailed quantitative analysis of this runtime vs. accuracy trade-off is provided in next section.

#### 4.4.2. Computational Runtime Comparison

The computational analysis in Table 5 highlights several key aspects of our framework. First, SfM is a mandatory common pre-requisite for all visual localization pipelines, as it establishes the initial sparse geometric backbone. In our experiments, the SfM sanitization process follows the methodology and datasets described in [27] to ensure precise camera-to-scene alignment. Furthermore, while the execution of LEGS remains efficient, the subsequent training phase (LEGS+SeqACE) inevitably incurs additional time due to the expanded scale of the augmented dataset. This represents a deliberate trade-off between offline computational budget and localization accuracy; we prioritize the latter to bridge the robustness gap in sparse-view environments. Given that this training is performed offline, the significant gains in pose estimation accuracy justify the increased training duration.

#### 4.4.3. Cross-Method Generalizability and Backbone Ablation

To validate the model-agnostic benefits of the LEGS framework, we conduct a comprehensive ablation study across four representative localization architectures: APR-based models (PoseNet [1], MS-Transformer [29]) and SCR-based models (ACE [4], SeqACE [5]). As LEGS is a data-centric augmentation module, it should theoretically enhance any pose-learning backbone by minimizing the geometric discrepancy between the training and test distributions.

As summarized in Table 6, LEGS provides consistent performance gains across all tested backbones and scenes, confirming its versatility. However, an interesting trend emerges: the relative improvement rate (%) tends to decrease as the baseline performance of the backbone increases. We attribute this to the fact that models with stronger baseline performance—achieved even with identical sparse training data—possess superior inherent generalization capabilities toward unseen test views. In this context, modern state-of-the-art models can be viewed as having an internal mechanism that mimics the benefits of training on a denser viewpoint distribution, such as through robust geometric priors or temporal sequence modeling.

Nevertheless, even for these high-performance models, LEGS acts as a critical precision-booster. While SCR-based methods show a more refined absolute accuracy (reaching the 10–20 cm range) compared to APR models, it is the synergistic effect of LEGS’s sanitized SfM and SE(3)-aware densification that allows them to bridge the final gap to peak precision.

#### 4.4.4. Sensitivity Analysis of SE(3) NMS Thresholds

To evaluate the impact of the NMS thresholds ($\sigma_{t},\sigma_{r}$) on the quality of the augmented training set, we conducted a sensitivity analysis as summarized in Table 7. The NMS process is designed to ensure a blue-noise-like spatial distribution; however, the choice of thresholds involves a critical trade-off between viewpoint diversity and data redundancy.

When the translation threshold is set too aggressively (e.g., $\sigma_{t}=0.5$ m), the framework prunes valid spatial samples, preventing the localizer from learning fine-grained positional cues and leading to increased translation errors. Similarly, an excessive rotation threshold (e.g., $\sigma_{r}=30{}^{\circ}$) suppresses necessary angular diversity, significantly degrading rotation accuracy as the model fails to capture viewpoint-dependent feature variations. Conversely, when the thresholds are set too loosely (e.g., $\sigma_{t}=0.05$ m, $\sigma_{r}=5{}^{\circ}$), the number of synthesized images increases tremendously compared to our default setting. However, as shown in Table 7, this surge in data volume yields negligible improvements in localization accuracy, as the redundant views offer little additional information to the image encoder. These results justify our default parameters ($\sigma_{t}=0.2$ m, $\sigma_{r}=15{}^{\circ}$) as an optimal “sweet spot” that maximizes geometric coverage while maintaining computational efficiency for training.

#### 4.4.5. Quantitative Viewpoint Coverage Analysis

To provide objective evidence of the coverage provided by LEGS, we analyze the spatial and viewpoint densification for the in-house dataset, as summarized in Table 8. The trends observed in Table 8 closely mirror the localization improvements reported in Table 3.

Fundamentally, the performance of a visual localization system is highly contingent upon the availability of training images that reside in close proximity to the test-time query poses. By employing LEGS, we significantly increase the probability of including such critical viewpoints in the training distribution. As shown in Table 8, our framework ensures that for each test query, the distance to the nearest training/virtual camera (within a 15° angular threshold) is substantially reduced compared to the baseline. This indicates that LEGS successfully “populates” the spatial gaps of sparse captures, generating virtual cameras that are more closely aligned with the actual test distribution. Consequently, this reduced spatial discrepancy between training and testing viewpoints serves as the primary driver for the enhanced pose estimation accuracy observed in our framework.

## 5. Conclusions

In this work, we presented LEGS, a data-centric, trajectory-agnostic framework that addresses the fundamental challenge of viewpoint imbalance in visual localization. By integrating sanitized SfM reconstruction with SE(3)-aware virtual camera generation guided by 3DGS, LEGS provides a robust training distribution that mitigates the inherent limitations of sparse or unordered captures. Our evaluations on the Cambridge Landmarks and in-house datasets indicate that principled viewpoint selection—prioritizing geometric coverage and visibility—serves as a significant contributing factor to the observed improvements in pose accuracy within our framework. While these results highlight the utility of LEGS as a versatile enhancement tool, further studies across a broader range of localization backbones will continue to validate the generalizability of this selection strategy. Future research will focus on evolving the current heuristic selection into a more principled paradigm, specifically investigating whether a lightweight learned scoring or ranking model can refine or replace the handcrafted pipeline to adaptively determine optimal viewpoints. This will involve scaling the framework to large-scale city-level environments and exploring the integration of differentiable rendering to further optimize the viewpoint proposal stage within an end-to-end learning paradigm. This approach will facilitate a more seamless coupling between data augmentation and the specific inductive biases of diverse localization architectures, enabling broader validation of our selection strategy. Furthermore, we aim to extend LEGS toward online or multi-sensor localization, effectively bridging the gap between offline reconstruction and real-time autonomous navigation in complex, dynamic environments.

While our experiments demonstrate performance gains under standard conditions, we acknowledge the challenges posed by extreme appearance variations, such as seasonal changes and dynamic objects. Although the Cambridge Landmarks dataset includes natural lighting variation and motion blur typical of outdoor handheld captures, our current framework primarily assumes static environments to ensure the stability of the 3DGS-based geometric proxy. We believe that the integration of the aforementioned lightweight learned ranking models could serve as a key mechanism to address these limitations by prioritizing views that are invariant to environmental noise. This will be a primary focus of our future work as we extend LEGS to handle even more unconstrained, in-the-wild scenarios.

**Figure 4:**
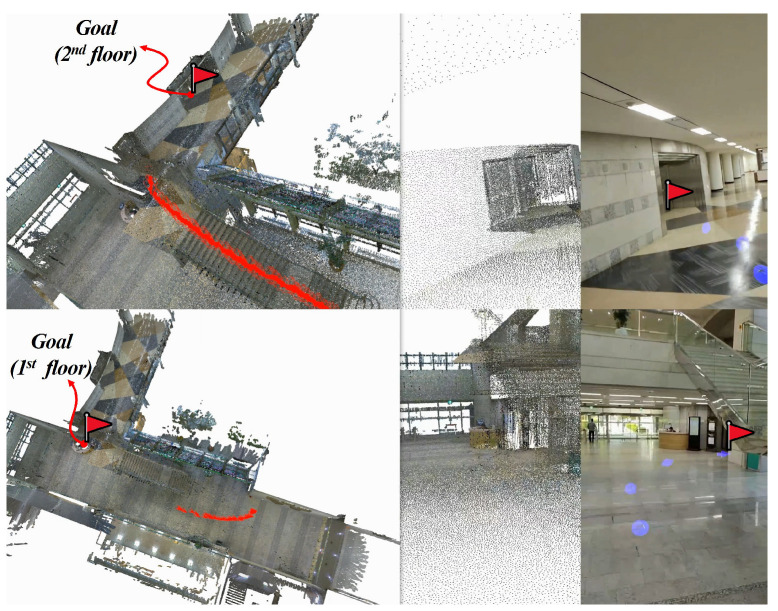
Demonstration of indoor navigation using our proposed LEGS-based visual localization framework. **Left:** estimated camera poses (red) visualized in reconstructed 3D space. **Center:** corresponding camera view rendered within the 3D environment. **Right:** captured real image and the navigation path toward the target location, indicated by blue arrows.

Beyond quantitative improvements, the framework’s design offers clear practical advantages. As illustrated in Figure 4, the improved localization stability achieved by LEGS enables reliable navigation and pose estimation in GPS-denied environments, facilitating applications in AR localization, vision-based indoor navigation, and mobile robotics. Because LEGS is modular and compatible with both structure-based and learning-based localization methods, it can serve as a plug-and-play augmentation module in broader perception and mapping systems.

## Figures and Tables

**Table 1:** Median localization error (translation in cm/rotation in degrees) for the Cambridge Landmarks dataset [1]. The downward arrow indicates lower values are better.

Scene	Median Error (cm/°) ↓					
	LENS (2021 [7])	DSAC++ (2018 [18])	DSAC* (2021 [19])	ACE (2023 [4])	SeqACE (2024 [5])	LEGS (Proposed)
King’s College	33/0.5	23/0.4	18/0.3	28/0.4	25/0.4	23/0.5
Shop Facade	27/1.6	9/0.4	5/0.3	5.7/0.3	5.7/0.3	5.4/0.3
St Mary’s Church	53/1.6	20/0.7	15/0.6	18/0.6	17/0.6	17/0.5
Old Hospital	44/0.9	24/0.5	21/0.4	31/0.6	27/0.6	27/0.5

**Table 2:** Scene statistics of our in-house dataset used for visual localization and 3DGS training. $n_{\mathrm{train}}$ and $n_{\mathrm{test}}$ denote the number of real training and testing images used for visual localization, where $n_{\mathrm{train}}$ also corresponds to the number of images used for 3DGS training. $n_{v}$ indicates the number of virtual cameras generated by our LEGS framework, and $\left(n_{\mathrm{train}}+n_{v}\right)$ represents the total number of training images for visual localization. Spatial dimensions are shown in meters. Note that 2D area values correspond to nearly planar spaces with negligible height variation, whereas the Stairs scene involves a multi-level structure, and thus height is explicitly included.

Scene	$n_{\mathrm{train}}$	$n_{\mathrm{test}}$	$n_{v}$	Size (m)
Show Room	411	102	1440	36.6 × 33.2
Testbed	605	151	1520	16.2 × 14.4
Conference Room	765	191	1980	49.8 × 36.8
Stairs	704	176	1210	121 × 93.5 × 15.7

**Table 3:** Median localization error (translation in cm/rotation in degrees) for our in-house dataset. The downward arrow indicates lower values are better.

Scene	Median Error (cm/°) ↓			
	SeqACE (2024 [5])	LEGS (Sanitization Only)	LEGS (Grid Only)	LEGS (Proposed)
Show Room	28/3.7	25/3.7	25/3.4	13/2.5
Testbed	26/4.4	23/4.2	19/4.1	10/2.1
Conference Room	48/5.4	40/5.1	39/4.4	23/3.5
Stairs	28/3.3	28/3.3	19/1.8	18/1.5

**Table 4:** Median localization error (translation in cm/rotation in degrees) for our in-house dataset for different synthetic images. The downward arrow indicates lower values are better.

Scene	Median Error (cm/°) ↓			
	Baseline (Real-Only)	$0.5n_{v}$	$n_{v}$	$2n_{v}$
Show Room	28/3.7	16/3.4	13/2.5	13/2.5
Testbed	26/4.4	13/4.2	10/2.1	10/2.0
Conference Room	48/5.4	32/5.1	23/3.5	22/3.4
Stairs	28/3.3	23/3.2	18/1.5	18/1.5

**Table 5:** Computational runtime comparison across our real-world indoor dataset. Training time is optimized and estimated based on a high-throughput training configuration (approx. 200 images/hour).

Scene	Runtime				
	SfM	LEGS (Stage 1) (Section 3.1)	LEGS (Stage 2) (Section 3.2)	SfM +SeqACE	LEGS +SeqACE
Show Room	1 h 47 min	2 h 31 min	0 h 15 min	4 h 05 min	9 h 18 min
Testbed	2 h 45 min	3 h 23 min	0 h 22 min	6 h 12 min	10 h 40 min
Conference Room	4 h 16 min	5 h 01 min	0 h 28 min	7 h 55 min	14 h 12 min
Stairs	3 h 05 min	3 h 58 min	0 h 13 min	5 h 38 min	9 h 53 min

**Table 6:** Cross-method ablation study on the in-house dataset. We report median translation/rotation error (cm/°). Translation errors are rounded to the nearest integer for clarity. The downward arrow indicates lower values are better.

Paradigm	Backbone	Scene	Baseline ↓	w/ LEGS ↓
APR	PoseNet [1]	Show Room	44/7.6	23/3.9
		Testbed	38/8.4	19/4.4
		Conference	63/10.8	32/5.4
		Stairs	46/6.9	25/2.7
	MS-Trans. [29]	Show Room	38/6.4	18/3.2
		Testbed	34/7.2	15/3.6
		Conference	56/8.6	27/4.2
		Stairs	39/5.5	22/2.2
SCR	ACE [4]	Show Room	32/4.7	15/2.7
		Testbed	30/5.4	13/2.9
		Conference	51/6.6	25/3.9
		Stairs	32/4.1	20/1.7
	SeqACE [5] (Ours)	Show Room	28/3.7	13/2.5
		Testbed	26/4.4	10/2.1
		Conference	48/5.4	23/3.5
		Stairs	28/3.3	18/1.5

**Table 7:** Ablation study on SE(3) NMS thresholds for our in-house dataset. Results are reported as median error (cm/°).

NMS Setting	Show Room	Testbed	Conference Room	Stairs
Aggressive Spatial ($\sigma_{t}=0.5$ m, $\sigma_{r}=15{}^{\circ}$)	19/2.8	16/2.3	31/4.1	24/2.0
Aggressive Angular ($\sigma_{t}=0.2$ m, $\sigma_{r}=30{}^{\circ}$)	15/4.2	12/3.8	26/5.5	20/3.1
Default ($\sigma_{t}=0.2$ m, $\sigma_{r}=15{}^{\circ}$)	13/2.5	10/2.1	23/3.5	18/1.5
Loose/Redundant ($\sigma_{t}=0.05$ m, $\sigma_{r}=5{}^{\circ}$)	12/2.4	10/2.0	22/3.4	17/1.5

**Table 8:** Spatial and viewpoint densification statistics for the in-house dataset. The upward arrow indicates that higher values are better, while the downward arrow indicates that lower values represent better performance. Train and LEGS densities are defined as the number of images per m^3^. The “Avg. Dist” columns represent the mean distance from each test camera to its nearest neighbor satisfying Δrot<15° and Δdist<0.5 m.

Scene	Density (img/m^3^) ↑		Densification	Avg. Dist to NN (m) ↓	
	Train	LEGS		Train	LEGS
Show Room	0.34	1.52	4.47×	0.29	0.11
Testbed	2.59	9.11	3.52×	0.22	0.09
Conference Room	0.42	1.50	3.57×	0.40	0.18
Stairs	0.06	0.17	2.83×	0.26	0.15

## Data Availability

The Cambridge Landmarks presented in this study are openly available in King’s College scene at https://www.repository.cam.ac.uk/items/53788265-cb98-42ee-b85b-7a0cbc8eddb3 (accessed on 20 October 2025), Shop Facade scene at https://www.repository.cam.ac.uk/items/39c82694-0e6d-487c-ac85-a649ee3bdca7 (accessed on 20 October 2025), Old Hospital scene at https://www.repository.cam.ac.uk/items/dc0a85ed-5986-42e0-8cf5-b5d200dc6c45 (accessed on 20 October 2025), St Mary’s Church scene at https://www.repository.cam.ac.uk/items/70c63f92-5a21-4390-b211-f044a961e36e (accessed on 20 October 2025). The in-house dataset is proprietary and cannot be released due to privacy and security restrictions.

## Abbreviations

The following abbreviations are used in this manuscript:

SfM	Structure-from-motion
NVS	Novel View Synthesis
NeRF	Neural Radiance Fields
3DGS	3D Gaussian Splatting
DoF	Degree-of-freedom
AR	Augmented Reality
APR	Absolute pose regression
SCR	Scene coordinate regression
NMS	non-maximum suppression
